# Primary Amoebic Meningoencephalitis Caused by *Naegleria fowleri*: An Old Enemy Presenting New Challenges

**DOI:** 10.1371/journal.pntd.0003017

**Published:** 2014-08-14

**Authors:** Ruqaiyyah Siddiqui, Naveed Ahmed Khan

**Affiliations:** Department of Biological and Biomedical Sciences, Aga Khan University, Karachi, Pakistan; Georgetown University, United States of America

## Abstract

First discovered in 1899, *Naegleria fowleri* is a protist pathogen, known to infect the central nervous system and produce primary amoebic meningoencephalitis. The most distressing aspect is that the fatality rate has remained more than 95%, despite our advances in antimicrobial chemotherapy and supportive care. Although rare worldwide, most cases have been reported in the United States, Australia, and Europe (France). A large number of cases in developing countries go unnoticed. In particular, religious, recreational, and cultural practices such as ritual ablution and/or purifications, Ayurveda, and the use of neti pots for nasal irrigation can contribute to this devastating infection. With increasing water scarcity and public reliance on water storage, here we debate the need for increased awareness of primary amoebic meningoencephalitis and the associated risk factors, particularly in developing countries.

## Purpose

Despite advances in the antimicrobial chemotherapy and supportive care, the fatality rate associated with primary amoebic meningoencephalitis (PAM) due to *Naegleria fowleri* has remained over 95% [1]–[3]. In part, this is due to the lack of availability of effective antimicrobial compounds that can target *N. fowleri* residing in the brain tissue. Religious, recreational, and cultural practices such as ritual ablution and/or purifications, Ayurveda, and the use of neti pots for nasal irrigation leading to contracting *N. fowleri* have highlighted PAM as an important threat to human health [4]–[6]. With the devastating nature of this disease and problems associated with its chemotherapy, the overall aim of this article is to discuss PAM-associated risk factors with an eye to advocate preventative strategies.

## Methods

A PubMed search using “*Naegleria fowleri*” combined with “Primary amoebic meningoencephalitis,” “ablution,” “epidemiology,” “diagnosis,” “pathogenesis,” “nasal irrigation,” and “neti pots” as keywords was carried out. In addition, we consulted conference proceedings, original unpublished research undertaken in our laboratories, and discussions in specific forums (e.g., the Free-Living Amoebae Meetings held in 2005 in Ceské Budejovice, Czech Republic; 2007 in Wako City, Japan; 2009 in Tenerife, Spain; 2011 in Jamaica, West Indies; and 2013 in Vienna, Austria).

## What Is Primary Amoebic Meningoencephalitis Due to *N. fowleri*?

Primary amoebic meningoencephalitis due to the protist pathogen *N. fowleri* is an acute, fulminant, necrotizing, and hemorrhagic meningoencephalitis, characterised by severe headache, stiff neck, fever (38.5°C–41°C), altered mental status, seizures, and coma, leading almost always to death [7]–[10]. *N. fowleri* invades humans via intact or disrupted nasal mucosa, crosses the cribriform plate, migrates along the basilar brain from the olfactory bulbs and tracts to the cerebellum, deeply penetrates the cortex to the periventricular system, and incites meningoencephalitis with rapid cerebral edema, resulting in cerebellar herniation. The olfactory bulbs and orbitofrontal cortices are necrotic and haemorrhagic. Histology has shown acute inflammatory reaction, mainly composed by neutrophils with extensive areas of lytic necrosis with the presence of several trophozoites. The literature in human and in experimental mouse models showed that the fibrinopurulent exudate is practically absent [11], [12]. Increased intracranial pressure and herniation are usually the cause of death. In the advanced stage, the red blood cells increase up to 24,600 per mm^3^. The white blood cell count (predominantly polymorphonuclear leukocytes [PMN]), varies from 300 cells per mm^3^ to as high as 26,000 per mm^3^. The protein concentration ranges from 100 mg to 1000 mg per 100 mL, and glucose may be 10 mg per 100 mL or lower [2], [7], [10]. The rapid diagnosis is generally achieved through microscopic examination of freshly drawn cerebrospinal fluid (CSF), to visualize motile *N. fowleri*. Trophozoites can be identified by Giemsa or Wright stains of CSF smears combined with the enflagellation test. These two methods could be enough at the beginning, since a rapid diagnosis is necessary, to start an opportune treatment. The immunofluorescence assay using anti–*N. fowleri* antibody [13], culture of amoebae by placing CSF on non-nutrient agar plate seeded with bacteria as a food source [2], PCR-based molecular methods [14], and the use of *in vitro*, *ex vivo* and *in vivo* animal models [15], [16] are useful methods to corroborate the diagnosis; however, they are time consuming. The scans of the brain show obliteration of the cisternae around the midbrain and the subarachnoid space over the cerebral hemispheres. Marked diffuse enhancement in these regions may be seen after administration of intravenous contrast medium [2], [7]. The drug of choice is the antifungal polyene, amphotericin B; however, the disease prognosis remains extremely poor (∼98% fatality rate). The incubation period from exposure leading to meningoencephalitis may range from one to 16 days [3], [7], [9]. Although PAM infections had occurred in Virginia in 1937 [17], the first reported infection of PAM was described in 1965 in Australia [18]. To date, a few hundred cases of PAM have been reported worldwide, with most cases reported in the United States, Australia, and Europe (France).

## Risk Factors: Past and Present

Evidently, deaths due to *N. fowleri* are on the rise [3], [8], [10]. In the past, PAM cases were reported in the developed countries in people who swim in fresh water during the hot summer months [7], [8]. However, prolonged hot and dry periods due to global warming are causing higher freshwater temperatures that are coinciding with augmented amoebal densities in water supplies, as well as an increase in recreational activities that are likely attributing to a rise in PAM cases [4]–[6],[19],[20]. More recently, it is reported that PAM infections are claiming the lives of young men in developing countries, such as Pakistan [4], [5]. Although recreational activities are likely attributing to PAM cases, reports from Pakistan suggest that the victims habitually did not have a history of swimming but were yet succumbing to this deadly infection [4], [5]. Thus there is a need to revisit risk factors that can contribute to PAM and debate possible preventative strategies.

### Recreational activities

**Figure 1:**
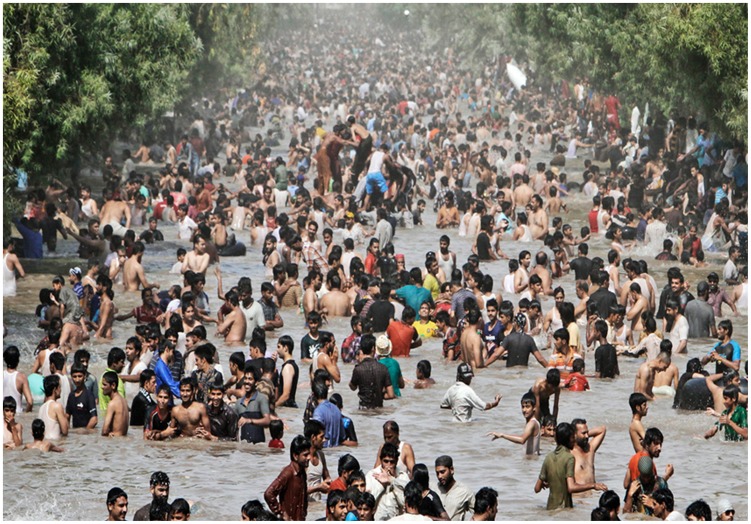
Thousands of people can be seen swimming in the canal that passes through the city of Lahore, without nearby facilities for defecation and urination.

**Figure 2:**
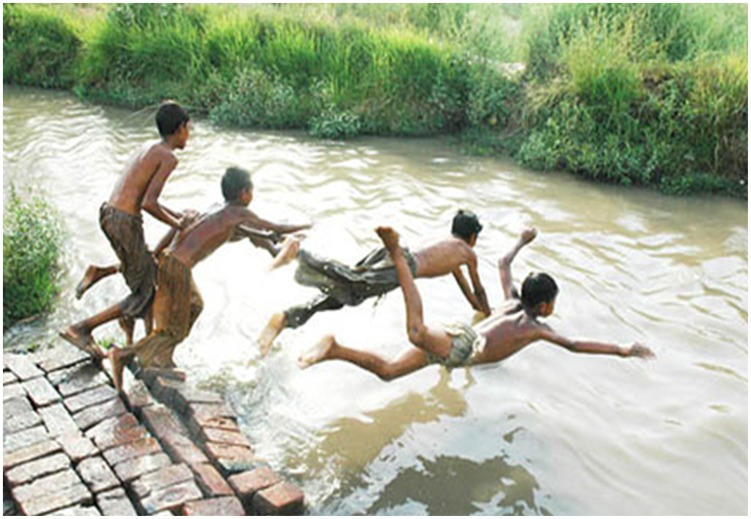
Children swimming in untreated water in the small village nearby Lahore. The participants had no concerns of their photographs being taken and potentially published.

**Figure 3:**
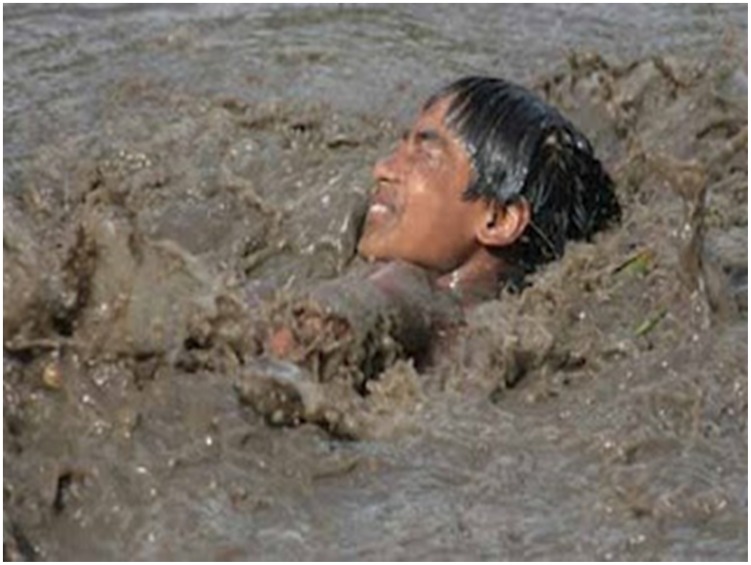
Swimming in polluted water is a routine activity, especially during summer months. The participant had no concerns of their photographs being taken and potentially published.

Being a free-living amoeboflagellate, *N. fowleri* thrives in freshwater, including freshwater lakes, river, canals, geothermal springs, spas, untreated domestic water supplies and swimming pools, warm water discharges from electrical power plants, etc. [1], [8], [21]–[28]. *N. fowleri* feeds on bacteria and organic debris in freshwater and exists in three life forms, the environmentally stable cyst form, the motile amoeboid-form referred to as the trophozoite form, and a flagellate form. These so-called “brain eating amoebae” invade the nervous system via the nose, when contaminated water is deeply inhaled into the nostrils during recreational activities. For example, jumping in water without a nose clip may force amoebae-containing water into the nostrils. Amoebae migrate along the olfactory neuroepithelial route to the brain tissue, where they cause severe haemorrhaging and inflammation, resulting in widespread brain tissue destruction within days [7], [8]. Historically, these infections have been reported from developed countries and preventative measures have included campaigns for increased awareness among the public as well as medial professionals. Although not a single PAM case associated with recreational activity is reported from Pakistan, our recent visits to large cities and small villages suggest that PAM is likely rampant in this part of the world. With temperatures reaching up to 50°C, while water temperatures are recorded at 30–35°C, and prolonged power cuts, millions of people turn to freshwater canals, ponds, standing water etc. For example, in Lahore alone (one of the largest cities in Pakistan) thousands of people can be seen swimming in the canal that passes through the city (Figure 1). The crowds are neverending and can be seen for miles along the canal, and this “recreational activity” goes on almost every day for months during the summer period. Lack of available toilet facilities and use of same waters to defecate with no apparent signage for potential dangers of swimming is both noticeable and disturbing. The presence of *N. fowleri* in these waters [29], lack of awareness and/or control measures, poor healthcare infrastructure, and unavailability of effective drugs to counter this infection present a major health hazard for the community. The question arises that if large cities are unable to identify and counter such a threat, the situation must be dire in poor communities in small villages that constitute more than 70% of the population (Figure 2). With power cuts reaching close to 20 hours per day, sometimes the only recreational activity available to young men and children in hot summer weather is swimming in the contaminated water (Figure 3). The countless stories of sudden deaths of young people in these communities, unwillingness of the majority of families to allow post-mortem autopsies, as well as lack of available expertise to identify causative agent, lack of access to medical facilities, and poorly equipped hospitals, along with the potential threat of drug-resistant *N. fowleri* transmission, suggest the need for vigilance.

### Religious practices

**Figure 4:**
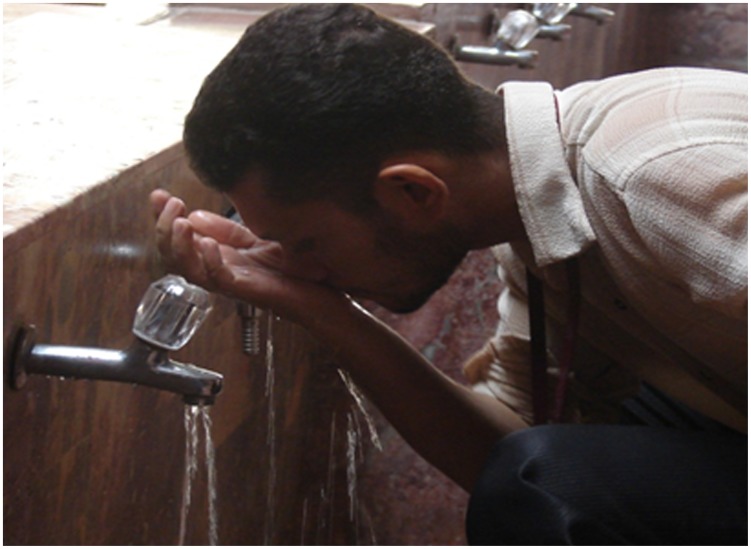
Ablution involves nasal cleansing. The use of contaminated water together with forceful pushing up the nostrils, even though it is not required as part of the ablution practice, can lead to amoeba entry into the brain.

**Figure 5:**
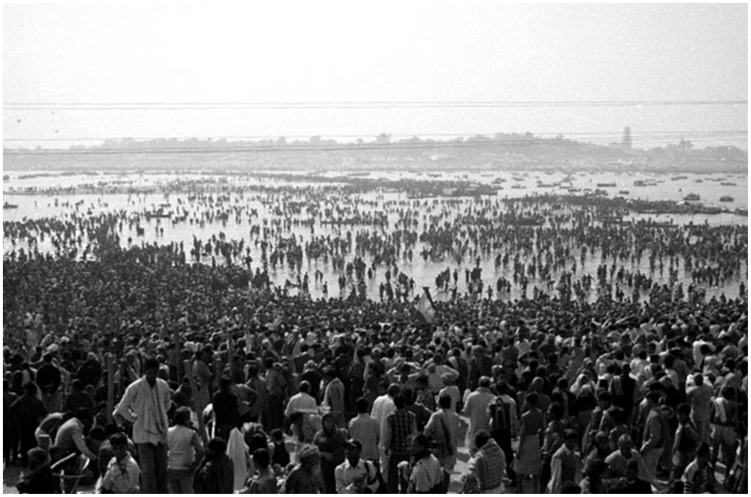
Millions of Hindus gather in the Indian city of Allahabad for a ritual bath in the sacred Ganges River.

In recent years, the Aga Khan University Hospital in Karachi (a leading private hospital in Pakistan) has seen a noticeable number of deaths due to PAM (approximately 20 per year) [5]. When investigating the possible risk factors (typically swimming in fresh waters), it was puzzling that none of the victims had a history of swimming. Despite being a cosmopolitan city, all victims generally are young men who strictly practice Islam. In addition to the loss of life, it is devastating for the affected family as often these men are the only breadwinners of the family. Muslims pray five times a day. Before every prayer, they perform ablution for cleansing. This involves washing the hands, mouth, nose, ears, face, arms and feet. When cleaning the nose, many people push water forcefully up the nostrils, even though this is not a mandatory part of the ablution process (Figure 4). Although the ablution practice has tremendous health benefits, it can only serve its purpose if water supplies are free of pathogenic microbes. In the presence of *N. fowleri* and if water is pushed up the nose, the amoebae access the nasal mucosa to invade the brain and cause this lethal infection. Recently, the first death of a Muslim male patient performing ablution in the United States has been reported [30]. This suggests that rigorous ablution is an important risk factor in contracting PAM. Thus there is a need for awareness to take measures to make water safer for ritual nasal rinsing. Using sterile water that is boiled for at least one minute and left to cool, or water filtered to remove small organisms, or water disinfected appropriately using recommended concentrations of chlorine together with careful ablution (not pushing water inside nostrils vehemently) should minimize the risk in contracting PAM infection [30]. Notably, Aga Khan University Hospital (AKUH) is a small, private hospital and the large population of the city (∼23 million) relies on public hospitals, yet not a single PAM case has been reported from any hospital in Pakistan except AKUH. Given that we are witnessing such a high number of deaths at the AKUH, the situation must be dire in the rest of the country, in the villages accounting for more than 70% of the population and with no or limited access to poorly equipped hospitals. Other religious festivals such as the Kumbh Mela, where millions of Hindus gather in the Indian city of Allahabad for a ritual bath in the sacred Ganges River pose a risk to public health in the transmission of infectious agents (Figure 5). In this month-long festival, the bathing takes place in an area known as the Sangam at the confluence of the Ganges and Yamuna rivers and a third mythical waterway called the Saraswati, and up to 100 million people participate in this holy bathing festival [19]. At present, there is neither report of PAM-associated with this practice nor the prevalence of *N. fowleri* in these waters, and this should be investigated in future studies.

## Therapeutic Interventions

**Figure 6:**
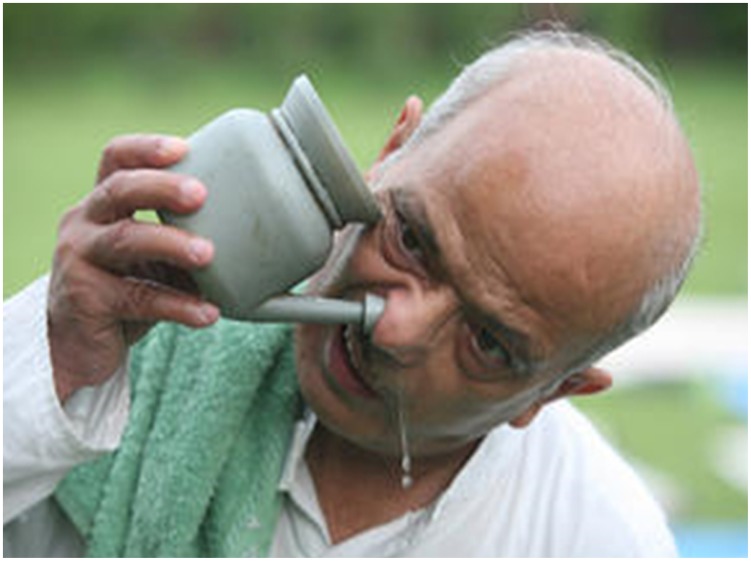
Nasal cleansing/irrigation using neti pots can provide relief to patients with sinusitis by flushing out excess mucus and debris from the nose. These are routine activities and participants had no concerns of their photographs being taken and potentially published.

Nasal cleansing/irrigation using neti pots can provide relief to patients with sinusitis including symptoms of facial pain, headache, cough, rhinorrhea (allergic rhinitis) and nasal congestion. Routine nasal cleansing can reduce medication used by patients with sinusitis and provide relief for hay fever, common cold, and other chronic sinus and nasal symptoms [31], [32]. The basis of such an adjunct therapy is that the nasal cavity is washed to flush out excess mucus and debris from the nose and sinuses and moisten the mucous membranes. It is recommended that nasal irrigation be performed using saline solution (0.9% non-iodized sodium chloride in purified or filtered warm water, with or without inclusion of a buffering agent such as sodium bicarbonate) [33], [34]. In addition, nasal irrigation is used in practices such as Ayurveda, also known as “jala neti,” which involves sniffing water from cupped hands and then blowing it out. Nasal irrigation is performed using a device shaped like Aladdin's lamp that is filled with saline. The water flows out the tip of the pot into one nostril. Gravity takes the water around the back of the nostril and drains out the opposite side of the nose (Figure 6). Then the same procedure is repeated on the opposite side. Although nasal irrigation promotes good sinus and nasal health, it can only be effective if purified, filtered, or boiled water is used. The use of unboiled or otherwise unsterilized water has proven to be an important risk factor in contracting PAM [6], [35]. Given the widespread use of this practice globally and the lack of availability of clean (sterilized/filtered) water to the majority of the population in the developing countries, it is likely that a large number of PAM go unnoticed. This highlights the importance of raising awareness about this disease among physicians as well as the community.

## Host Factors

The majority of PAM cases have been reported in young males with a history of exposure to contaminated water. Considering recreational activities, this could be logically explained by fervent involvement of young men in outdoor activities. With regards to ablution, all PAM cases at AKUH have been observed in young males, and not a single case has been reported in women. Although the virulent nature of *N. fowleri* combined with rigorous ablution practices by males may be a contributing factor, it is likely that other predisposing factors may play a role in contracting PAM infection. Thus there is need to reveal causes, be they (i) genetic, (ii) biochemical changes, (iii) underlying disease prerequisite leading to damaged or abnormal mucosa (containing abnormal levels of immune factors), and/or (iv) rigorous, routine nasal irrigation. Future research is needed to address these issues. Given the rapid onset and progression of PAM in humans and the route of infection, future studies are needed to determine precise host factors to help develop preventative strategies and/or therapeutic interventions for susceptible hosts.

## Unsafe Water Sources and Ablution: What Choice Is There?

Developing countries, such as Pakistan, face severe water shortage and there is excessive public reliance on water storage tanks and wells. Unfortunately, these tanks are breeding grounds for the propagation of free-living amoebae as well as other microbial communities. Surprisingly, in spite of concerns about amoebic pathogens, there have been very few reports of central nervous system (CNS) infections due to free-living amoebae in the developing countries [5]. The only available data is from the Aga Khan University Hospital in Karachi, Pakistan that has witnessed a surge in the number of deaths reported in 2008 from 13 to more than 20 in 2010 [4], [5]. Almost all the cases reported had a history of exposure to tap water that they had used for ablution, suggesting that patients who succumbed to this infection were using *N. fowleri*–contaminated tap water [4], [5], [29]. The potential of these organisms to cause serious infections makes it imperative to investigate the occurrence of these organisms in our environment. If we take Pakistan as an example, the country's population was 180.8 million in 2009, which is projected to reach 208 million by 2020 [36], [37]. Karachi has a metropolitan area population of 23 million people as a result of rapid urbanization in Pakistan. It is expected that more than 30,000 people (including 20,000 children) die every year in just Karachi alone due to unsafe water [36], [38]. The circumstances are further exacerbated by a relatively warm climate, which favours the growth of pathogenic free-living amoebae and other microbes. Recently, a study was carried out in Karachi, Pakistan to determine the presence and distribution of free-living amoebae (*Acanthamoeba* spp., *N. fowleri, and Balamuthia mandrillaris*) in drinking water supplies from different areas of the city. The results revealed that 38% of domestic drinking water samples tested positive for the presence of pathogenic free-living amoebae, of which 30% contained *Acanthamoeba* spp. and 8% contained *N. fowleri* [29]. The presence of these free-living amoebae in tap water could be due to poor water disinfection/management, old plumbing, poor tap water hygiene, environmental settings, and more importantly, the use of water storage tanks and/or wells. Standing water is reserved for long periods of time and stored because of water shortages; resulting in exposure to environmental microorgansims, some of which are potential pathogens such as *N. fowleri*. In turn, the stored water is used for nasal cleansing/irrigation as part of ablution and/or recreational activities or therapeutic interventions. The general public needs to be made aware of the health risks associated with the use of storage tanks. More importantly, the public needs to be informed of the appropriate maintenance of water storage tanks together with support, as occurrence of amoebae in domestic tap water supplies is an important public health risk. Apart from homes, inappropriate maintenance of water storage in mosques can also contribute to microbial contamination. As the majority of public perform ablution at mosques in preparation for prayers, it is imperative that water storage tanks are cleaned and disinfected as per World Health Organization guidelines [39]. Overall, it is likely that a vast number of PAM infections due to *N. fowleri* are undetected, and the actual burden is significantly higher, especially in developing countries such as Pakistan, which warrants further investigation.

## Conclusions

The increasing water shortage, poor water maintenance, heavy reliance on water storage wells or tanks where microbial communities thrive, and practices such as ablution and recreational activities or therapeutic interventions involving nasal irrigation can lead to PAM, resulting in devastating consequences. Rising temperatures in recent years due to global warming, together with poor infrastructure of wastewater management and sanitation as well as drug resistance will cause a further rise in the number of deaths due to infectious diseases in general and PAM in particular. There is an urgent need to increase awareness of the public as well as health professionals and for a mixture of educational and behavioral modification strategies. The use of nose clips should be encouraged to avoid any traumatic disruptions in the nasal mucosal linings during water-related activities in warm freshwater, such as lakes, rivers, ponds, bayous, and hot springs. When performing ablution, water that is boiled for one minute and left to cool, or filtered to remove small organisms, or disinfected appropriately should be utilized, together with cautious ablution (not pushing water inside nostrils vehemently), in order to avoid serious consequences for communities living in developing countries. In addition, there is a need to develop novel, cost-effective anti–*N. fowleri* compounds that can effectively cross the blood-brain barrier to target parasites residing deep in the brain tissue.

**Key Learning Points**

- Primary amoebic meningoencephalitis due to *Naegleria fowleri* is a fatal infection with a mortality rate of more than 95%, despite advances in antimicrobial chemotherapy and supportive care.
- Although considered rare, a large number of cases in developing countries go unnoticed.
- In particular, religious, recreational, and cultural practices such as ritual ablution and/or purifications, Ayurveda, and the use of neti pots for nasal irrigation can contribute to this devastating infection.
- With the devastating nature of this disease and problems associated with its chemotherapy, the overall aim of the present article is to focus on PAM-associated risk factors with an eye to advocate preventative strategies.

**Top Five Papers**

1. Marciano-Cabral F, Cabral GA (2007) The immune response to *Naegleria fowleri* amebae and pathogenesis of infection. FEMS Immunol Med Microbiol 51: 243–259.
2. Visvesvara GS, Moura H, Schuster FL (2007) Pathogenic and opportunistic free-living amoebae: *Acanthamoeba* spp., *Balamuthia mandrillaris*, *Naegleria fowleri*, and *Sappinia diploidea*. FEMS Immunol Med Microbiol 50: 1–26.
3. Diaz J (2012) Seasonal primary amebic meningoencephalitis (PAM) in the south: Summertime is PAM time. J La State Med Soc 164: 148–155.
4. De Jonckheere JF (2011) Origin and evolution of the worldwide distributed pathogenic amoeboflagellate *Naegleria fowleri*. Infect Genet Evol 11: 1520–1528.
5. Guarner J, Bartlett J, Shieh WJ, Paddock CD, Visvesvara GS, et al. (2007) Histopathologic spectrum and immunohistochemical diagnosis of amebic meningoencephalitis. Mod Pathol 20: 1230–1237.
